# Sex differences in prognosis factors in patients with lung cancer: A nationwide retrospective cohort study in Korea

**DOI:** 10.1371/journal.pone.0300389

**Published:** 2024-05-10

**Authors:** Youn Huh, Yeo Ju Sohn, Hae-Rim Kim, Hyejin Chun, Hwa Jung Kim, Ki Young Son

**Affiliations:** 1 Department of Family Medicine, Uijeongbu Eulji Medical Center, Eulji University, Gyeonggi-do, Republic of Korea; 2 Department of Family Medicine, Ewha Womans University College of Medicine, Seoul, Republic of Korea; 3 College of Natural Science, School of Statistics, University of Seoul, Seoul, Republic of Korea; 4 Departments of Preventive Medicine and Clinical Epidemiology and Biostatics, Asan Medical Center, University of Ulsan College of Medicine, Seoul, Republic of Korea; 5 Department of Family Medicine, Asan Medical Center, University of Ulsan College of Medicine, Seoul, Republic of Korea; Kyung Hee University School of Medicine, REPUBLIC OF KOREA

## Abstract

Large-scale studies elucidating sex differences in factors impacting prognosis and sex-specific prognossis factors scoring in patients with lung cancer are insufficient. The present study aimed to develop a model to predict sex-specific prognosis factors in Korean patients with lung cancer. This nationwide cohort study included 96,255 patients aged ≥19 years diagnosed with lung cancer and underwent Korean National Health Insurance Service health examinations between January 1, 2005 and December 31, 2015 and followed until 2020. Factors associated with prognosis were estimated using multivariable Cox proportional hazards regression analyses, and separate prognosis scores were calculated for male and female patients. The sex-specific risk scoring models were validated with Kaplan–Meier survival curves and c-statistic. During a mean follow-up of 2.8 years, 60.5% of the patients died. In male patients with lung cancer, age ≥ 65 years (24 points) had the highest mortality risk score, followed by chemotherapy in combination with radiotherapy (16 points), chemotherapy (14 points), and radiotherapy (11 points). In female patients with lung cancer, chemotherapy in combination with radiotherapy (19 points) had the highest mortality risk score, followed by chemotherapy (16 points), age ≥ 65 years (13 points), and radiotherapy (13 points). The analysis of patients categorized into three risk groups based on risk scores revealed that the fatality rates within 5 years were 7%, 54%, and 89% in the low-, intermediate-, and high-risk groups for male patients and 3%, 46%, 85% in the low-, intermediate-, and high-risk groups for female patients, respectively. The c-statistic was 0.86 for male patients and 0.85 for female patients. The strongest fatality risk factors in lung cancer were age ≥ 65 years in male patients and chemotherapy in female patients. The present study developed sex-specific prognosis scoring models to predict fatality risk in patients with lung cancer.

## Introduction

In 2020, lung cancer was estimated the second most common cancer in both men and women and the most common cancer in men worldwide [1]. In addition, lung cancer is the leading cause of cancer-related mortality globally, despite improvements in treatment approaches including surgery, radiotherapy, and chemotherapy [2]. In Korea, lung cancer is the third most common cancer, following gastric and thyroid cancers, and is the second common cancer in men and the fifth common cancer in women [3]. The most important cause of cancer mortality was lung cancer [3].

Several studies have reported mortality risk factors in patients with lung cancer [4,5]. One study reported age ≥ 75 years, male sex, radiotherapy, infection, and organ failure as important mortality risk factors in patients with lung cancer [4]. In addition, lung damage caused by radiotherapy was an important poor prognostic factor in patients with lung cancer [5]. In another study, the risk of mortality was higher in current smokers than in ex-smokers and never-smokers among patients with non-small-cell lung cancer [6]. Additionally, there are sex differences in the types of lung cancer, treatment methods, and prognosis, because of sex differences of smoking status [7].

Previous studies reported sex differences in prognosis among patients with lung cancer [8,9]. In addition, the causes of the sex difference in lung cancer prognosis include age, smoking status, genetic factors, and early lung cancer screening [10]. Survival with lung cancer was 1.5 times higher in women than in men, even after adjusting for several variables [8].

However, only a limited number of large-scale studies investigated sex differences in factors influencing lung cancer prognosis. Therefore, the present study investigated sex differences in factors associated with prognosis. The present study used a large-scale cohort database representative of South Korea and evaluated various variables such as lung cancer treatment methods, lifestyle factors, socioeconomics, comorbidities, and anthropometric measurements. In addition, the present study aimed to develop a predictive model of prognosis specifically in male and female patients with lung cancer.

## Methods

### Data source and study population

The present study used the Korean National Health Insurance Service (NHIS) database to retrospectively evaluate the nationwide cohort of patients with lung cancer. The NHIS was established in 2000 as a single universal insurer in South Korea and provides at least biennial health examinations for all insured South Koreans aged ≥19 years. Therefore, the NHIS retains an extensive medical database of demographic characteristics, health examinations, disease diagnoses, medical treatments, and procedures based on medical claims according to the International Classification of Diseases-10th (ICD-10) codes for nearly the entire South Korean population [11]. The NHIS data are accessible by qualified researchers who submit a study protocol approved by official review committees. We accessed the NHIS database between March 31, 2022 and December 10, 2022 for research purposes.

A total of 228,258 patients with lung cancer who underwent national health examinations by the NHIS between January 1, 2005 and December 31, 2015 were identified. Individuals with missing data for any of the study variables (n = 47,134) and those aged <19 years (n = 100) were excluded. Additionally, those who were not treated in Seoul, the biggest city of South Korea, were excluded because 16.6% of all Korean patients with lung cancer and 22.1% of patients who received treatment were in Seoul. Finally, 96,255 patients, including 65,816 male and 30,439 female patients, were eligible for the study. The study end date was December 31, 2020. The present study was approved by the Institutional Review Board of Uijeongbu Eulji Medical Center (approval no: UEMC 2021-08-022) and conducted according to the principles of the Declaration of Helsinki. The requirement for informed consent was waived for this study which used anonymized patient data.

### Study variables and outcomes

The NHIS database contains detailed information on demographic and lifestyle characteristics, which are captured using standardized self-administered questionnaires. Patients in the lowest 10% of the income range were classified as those with low income, and the remaining patients were considered as the non-low-income category. Smoking status was classified as current smoker or non-smoker based on smoking history. Alcohol consumers were defined as individuals consuming >0 g alcohol per week. Regular physical activity was defined as high-intensity exercise for ≥3 days/week or moderate-intensity exercise for ≥5 days/week [12,13]. Health examinations were conducted by qualified medical staff and included anthropometric and laboratory measurements. Height, weight, and waist circumference were measured, and body mass index (BMI) was calculated as weight divided by height squared (kg/m^2^). Systolic and diastolic blood pressures were measured with the patient seated for at least 5 min. Blood samples were obtained after overnight fasting to determine serum glucose and total cholesterol concentrations. All demographic and lifestyle characteristics and anthropometric and laboratory measurements were evaluated as the last information before lung cancer diagnosis.

In the present study, 20 chronic diseases were included as comorbidities and identified using ICD-10 codes classified by the U.S. Office of the Assistant Secretary of Health [10]; and the patients were categorized into those with 0, 1, 2, 3, 4, or ≥5 comorbidities before lung cancer diagnosis. Treatment approaches for lung cancer were categorized as none, surgery, chemotherapy, radiotherapy, surgery in combination with chemotherapy, surgery in combination with radiotherapy, chemotherapy in combination with radiotherapy, and surgery in combination with chemotherapy and radiotherapy. Emergency room visits were defined as the presence of emergency visits within 1 year of death or December 31, 2020.

The primary study outcome was all-cause mortality during follow-up and included all eligible patients who were followed from baseline until the date of death or December 31, 2020, whichever came first.

### Statistical analysis

All statistical analyses were performed using the SAS software (version 9.4; SAS Institute, Cary, NC, USA) and the R package. Baseline characteristics categorized according to sex were reported as means ± standard deviation for continuous variables and numbers (percentages) for categorical variables. Continuous variables were compared using independent Student’s *t* test, and categorical variables using the chi-squared test. The incidence of all-cause mortality was calculated by dividing the number of events by 1000 person-years. Multivariable Cox proportional hazard regression analyses were performed to evaluate the association of covariates with the risk of all-cause mortality according to sex. All results were reported as hazard ratios (HRs) with 95% confidence intervals (CIs). In the present study, two models were used. Model 1 was not adjusted for any variables, whereas Model 2 was adjusted for all variables with a p value < 0.05 determined in Model 1. The adjusted variables were chosen which presented statistically significant differences in baseline characteristics and were showed to confound prognosis clinically and based on previous literatures. In Korea, all patients benefit from the National Health Insurance, and because patients only bear a very small portion of the cost for cancer diagnosis, the likelihood of treatment methods being determined by economic status is very low. In addition, risk factors identified by multivariate analyses were assigned weighted points proportional to their β regression coefficient values to develop a practical prognostic score. The study cohort was divided into the following three groups according to the sex-specific prognostic scores: low-risk (0–7 points), intermediate-risk (8–11 points), and high-risk (≥12 points). Kaplan–Meier survival curves of these three risk groups were generated to evaluate all-cause mortality risk. The predictive accuracy of the sex-specific scoring models was examined by calculating the c-statistic and evaluating the differenced in the probability of death within 5 years among the risk groups, which was calculated as (P_high_ − P_low_) / 100, where P_high_ was the predicted probability of death for a patient in the group with the worst prognosis and P_low_ was the predicted probability of death for a patient in the group with the best prognosis [14].

## Results

### Baseline characteristics of the study patients

Table 1 summarizes the baseline characteristics of 96,255 eligible patients with lung cancer categorized according to sex. The mean ages were 65.6 ± 10.9, 66.4 ± 10.4, and 63.9 ± 11.8 years in the overall cohort, male patients, and female patients, respectively. The prevalence of current smokers, alcohol consumers, and regular exercisers was higher in male patients than in female patients, whereas the prevalence of those with low income was higher in female patients than in male patients. The mean systolic and diastolic blood pressures were higher in male patients, whereas the mean body mass index and total cholesterol were higher in female patients. In both male and female patients, the higher the number of chronic diseases, the higher the prevalence, and the prevalence of those with ≥5 chronic diseases was higher in female patients than in male patients. Regarding treatment approaches, the rates of patients who received no therapy, those who received radiotherapy, and those who received radiotherapy in combination with surgery were higher in male patients than in female patients, whereas the rates of patients receiving other treatments were higher in female patients than in male patients. Finally, the prevalence of emergency room visits was higher in male patients than in female patients.

**Table 1:** Baseline characteristics of the study participants.

	Total	Sex		
		Male	Female	p value
	(N = 96,255)	(N = 65,816)	(N = 30,439)	
Age (years)	65.6 ± 10.9	66.4 ± 10.4	63.9 ± 11.8	<0.001
Low income	16,838 (17.5)	11,221 (17.1)	5,617 (18.5)	<0.001
Current smoker	26,339 (27.4)	24,991 (38.0)	1,348 (4.4)	<0.001
Alcohol drinker	18,869 (19.6)	17,927 (27.2)	942 (3.1)	<0.001
Regular exerciser	36,244 (37.7)	25,422 (38.6)	10,822 (35.6)	<0.001
BMI (kg/m^2^)	23.4 ± 3.1	23.3 ± 3.0	23.8 ± 3.3	<0.001
Systolic blood pressure (mmHg)	126.9 ± 16.5	127.5 ± 16.3	125.5 ± 16.8	<0.001
Diastolic blood pressure (mmHg)	77.2 ± 10.4	77.6 ± 10.3	76.3 ± 10.3	<0.001
Total cholesterol (mg/dL)	190.9 ± 43.2	186.4 ± 40.3	200.6 ± 47.4	<0.001
Number of chronic diseases				<0.001
0	2273 (2.4)	1657 (2.5)	616 (2.0)	
1	5940 (6.2)	4398 (6.7)	1542 (5.1)	
2	9203 (9.6)	6666 (10.1)	2537 (8.3)	
3	11,614 (12.1)	8431 (12.8)	3183 (10.5)	
4	12,820 (13.3)	9074 (13.8)	3746 (12.3)	
≥5	54,405 (56.5)	35,590 (54.1)	18,815 (61.8)	
Treatment of lung cancer				<0.001
None	33,927 (35.3)	23,641 (35.9)	10,286 (33.8)	
Chemotherapy	7652 (8.0)	4511 (6.9)	3141 (10.3)	
Surgery	15,381 (16.0)	9769 (14.8)	5612 (18.4)	
Radiation	16,092 (16.7)	13,118 (19.9)	2974 (9.8)	
Surgery + chemotherapy	2136 (2.2)	1137 (1.7)	999 (3.3)	
Surgery + radiation	4863 (5.1)	3,869 (5.9)	994 (3.3)	
Chemotherapy + radiation	12,358 (12.8)	7522 (11.4)	4836 (15.9)	
Surgery + chemotherapy + radiation	3846 (4.0)	2249 (3.4)	1597 (5.3)	
Emergence room visit (yes)	30,572 (31.8)	21,814 (33.1)	8758 (28.8)	<0.001

Abbreviation: BMI, body mass index.

Values are presented as means ± standard deviations or numbers (percentages).

### Risk scoring system based on factors associated with mortality risk in male patients with lung cancer

Table 2 summarizes the multivariable Cox regression analyses of factors associated with all-cause mortality in male patients with lung cancer, and Table 4 shows the risk scoring model for male patients with lung cancer based on these analyses. During a mean follow-up of 2.6 years, 65.9% (43,374) of the male patients died. Age ≥ 65 years (24 points) had the highest mortality risk score, followed by chemotherapy in combination with radiotherapy (16 points), chemotherapy (14 points), radiotherapy (11 points), emergence room visit (10 points), age between 45 and 64 years (8 points), current smoker (7 points), ≥5 chronic diseases (6 points), underweight (6 points), and 3–4 chronic diseases (4 points). However, surgery (−35 points) had the lowest mortality risk score, followed by surgery in combination with chemotherapy (−19 points), surgery in combination with radiotherapy (−15 points), surgery in combination with chemotherapy and radiotherapy (−10 points), regular exercise (−4 points), and obesity (−3 points).

**Table 2:** Multivariate Cox proportional hazard regression analyses of all-cause mortality in male patients with lung cancer.

Covariate	N	Death	Person-years	IR^a^	HR (95% CI)			
					Model 1^b^	P value	Model 2^c^	P value
Age (years)								
19–44	1919	814	6940	11.7	1 (reference)		1 (reference)	
45–64	23 784	12 642	75 219	16.8	1.39 (1.29–1.49)	<0.001	1.42 (1.32–1.52)	<0.001
≥65	40 113	29 918	87 618	34.2	2.61 (2.43–2.80)	<0.001	2.48 (2.31–2.67)	<0.001
Income								
Others	54 595	35 719	142 066	25.1	1 (reference)		1 (reference)	
Low	11 221	7655	27 711	27.6	1.08 (1.06–1.11)	<0.001	1.05 (1.03–1.08)	<0.001
Smoking status								
Non	40 825	25 166	113 455	22.2	1 (reference)		1 (reference)	
Current	24 991	18 208	56 323	32.3	1.38 (1.35–1.40)	<0.001	1.35 (1.32–1.37)	<0.001
Alcohol consumption								
No	47 889	31 037	126 038	24.6	1 (reference)		1 (reference)	
Yes	17 927	12 337	43 739	28.2	1.12 (1.10–1.14)	<0.001	1.08 (1.06–1.11)	<0.001
Regular exercise								
None	40 394	27 776	99 117	28.0	1 (reference)		1 (reference)	
Regular	25 422	15 598	70 660	22.1	0.81 (0.80–0.83)	<0.001	0.86 (0.85–0.88)	<0.001
BMI (kg/m^2^)								
<18.5	3488	2741	7033	39.0	1.38 (1.33–1.44)	<0.001	1.18 (1.13–1.22)	<0.001
18.5–25	43 587	29 276	109 954	26.6	1 (reference)		1 (reference)	
≥25	18 741	11 357	52 790	21.5	0.83 (0.81–0.85)	<0.001	0.90 (0.88–0.92)	<0.001
Number of chronic diseases								
0	1657	816	5506	14.8	1 (reference)		1 (reference)	
1	4398	2397	13 690	17.5	1.16 (1.07–1.26)	<0.001	1.05 (0.97–1.14)	0.196
2	6666	3964	19 227	20.6	1.35 (1.25–1.45)	<0.001	1.12 (1.04–1.20)	0.004
3	8431	5284	22 978	23.0	1.48 (1.38–1.60)	<0.001	1.18 (1.09–1.27)	<0.001
4	9074	5871	23 857	24.6	1.58 (1.46–1.70)	<0.001	1.21 (1.12–1.30)	<0.001
≥5	35 590	25 042	84 519	29.6	1.86 (1.73–1.99)	<0.001	1.30 (1.21–1.39)	<0.001
Treatment for lung cancer								
None	23 641	16 553	49 667	33.3	1 (reference)		1 (reference)	
Chemotherapy	4511	3836	9084	42.2	1.11 (1.07–1.15)	<0.001	1.09 (1.05–1.13)	<0.001
Surgery	9769	2184	41 905	5.2	0.17 (0.17–0.18)	<0.001	0.19 (0.18–0.19)	<0.001
Radiotherapy	13 118	10 930	23 939	45.7	1.23 (1.20–1.26)	<0.001	1.14 (1.11–1.17)	<0.001
Surgery + chemotherapy	1137	482	4606	10.5	0.33 (0.30–0.37)	<0.001	0.35 (0.32–0.38)	<0.001
Surgery + radiotherapy	3869	1883	13 802	13.6	0.43 (0.41–0.45)	<0.001	0.43 (0.41–0.45)	<0.001
Chemotherapy + radiotherapy	7522	6352	17 991	35.3	0.94 (0.91–0.97)	<0.001	1.02 (0.99–1.05)	0.286
Surgery + chemotherapy + radiotherapy	2249	1154	8783	13.1	0.41 (0.39–0.44)	<0.001	0.44 (0.42–0.47)	<0.001
Emergence room visit								
No	44 002	26 379	120 182	22.0	1 (reference)		1 (reference)	
Yes	21 814	16 995	49 595	34.3	1.41 (1.39–1.44)	<0.001	1.22 (1.20–1.25)	<0.001

Abbreviations: IR, incidence rate; HR, hazard ratio; CI, confidence interval; BMI, body mass index.

^a^ Incidence per 1000 person-years.

^b^ Model 1 was not adjusted for any variables.

^c^ Model 2 was adjusted for all variables with p value of <0.05 in Model 1.

### Risk scoring system based on factors associated with mortality risk in female patients with lung cancer

Table 3 shows the multivariable Cox regression analyses of factors associated with all-cause mortality in female patients with lung cancer. Table 4 shows the risk scoring model built based on these results. Briefly, 48.84% (14,867) of the female patients died during a mean follow-up of 3.4 years. Chemotherapy in combination with radiotherapy (19 points) had the highest mortality risk score, followed by chemotherapy (16 points), age ≥ 65 years (13 points), radiotherapy (13 points), emergence room visit (8 points), current smoker status (7 points), age between 45 and 64 years (3 points), and ≥5 chronic diseases (3 points). Conversely, surgery (−19 points) had the lowest mortality risk score, followed by surgery in combination with chemotherapy (−9 points), surgery in combination with radiotherapy (−6 points), surgery in combination with chemotherapy and radiotherapy (−3 points), regular exercise (−3 points), and obesity (−1 point).

**Table 3:** Multivariate Cox proportional hazard regression analyses of all-cause mortality in female patients with lung cancer.

Covariates	N	Death	Person-years	IR^a^	HR (95% CI)			
					Model 1^b^	P value	Model 2^c^	P value
Age (years)								
19–44	1619	594	6302	9.4	1 (reference)		1 (reference)	
45–64	13 644	5245	52 558	10.0	1.06 (0.97–1.15)	0.21	1.15 (1.05–1.26)	0.002
≥65	15 176	9028	43 944	20.5	2.09 (1.93–2.27)	<0.001	2.12 (1.94–2.32)	<0.001
Income								
Others	24 822	11 913	84 635	14.1	1 (reference)		1 (reference)	
Low	5617	2954	18 170	16.3	1.14 (1.10–1.19)	<0.001	1.10 (1.06–1.15)	<0.001
Smoking status								
Non	29 091	13 944	99 526	14.0	1 (reference)		1 (reference)	
Current	1348	923	3279	28.2	1.91 (1.79–2.04)	<0.001	1.58 (1.48–1.69)	<0.001
Alcohol consumption								
No	29 497	14 364	99 761	14.4	1 (reference)		1 (reference)	
Yes	942	503	3044	16.5	1.13 (1.04–1.24)	0.006	1.08 (0.99–1.18)	0.089
Regular exercise								
None	19 617	10 317	63 306	16.3	1 (reference)		1 (reference)	
Regular	10 822	4550	39 498	11.5	0.72 (0.70–0.75)	<0.001	0.81 (0.78–0.84)	<0.001
BMI (kg/m^2^)								
<18.5	1 184	628	3681	17.1	1.19 (1.10–1.29)	<0.001	1.09 (1.01–1.18)	0.037
18.5–25	19 241	9304	65 512	14.2	1 (reference)		1 (reference)	
≥25	10 014	4935	33 611	14.7	1.03 (1.00–1.07)	0.081	0.91 (0.88–0.95)	<0.001
Number of chronic diseases								
0		198	2519	7.9	1 (reference)		1 (reference)	
1	1542	614	5944	10.3	1.30 (1.11–1.52)	0.001	1.17 (1.00–1.38)	0.052
2	2537	1049	9500	11.0	1.38 (1.19–1.61)	<0.001	1.17 (1.01–1.36)	0.041
3	3183	1396	11 562	12.1	1.51 (1.30–1.75)	<0.001	1.20 (1.03–1.39)	0.019
4	3746	1638	13 421	12.2	1.52 (1.31–1.76)	<0.001	1.15 (0.99–1.34)	0.063
≥5	18 815	9972	59 859	16.7	2.05 (1.78–2.36)	<0.001	1.31 (1.14–1.52)	<0.001
Treatment of lung cancer								
None	10 286	4932	31 812	15.5	1 (reference)		1 (reference)	
Chemotherapy	3141	2426	7938	30.6	1.81 (1.73–1.91)	<0.001	1.70 (1.62–1.78)	<0.001
Surgery	5612	341	27 078	1.3	0.08 (0.08–0.09)	<0.001	0.09 (0.08–0.11)	<0.001
Radiotherapy	2974	2156	7021	30.7	1.85 (1.76–1.95)	<0.001	1.77 (1.68–1.86)	<0.001
Surgery + chemotherapy	999	288	4441	6.5	0.42 (0.38–0.48)	<0.001	0.43 (0.38–0.48)	<0.001
Surgery + radiotherapy	994	316	4102	7.7	0.50 (0.45–0.56)	<0.001	0.54 (0.48–0.61)	<0.001
Chemotherapy + radiotherapy	4836	3825	13 544	28.2	1.67 (1.60–1.75)	<0.001	1.78 (1.71–1.86)	<0.001
Surgery + chemotherapy + radiotherapy	1597	583	6868	8.5	0.55 (0.51–0.60)	<0.001	0.62 (0.57–0.67)	<0.001
Emergence room visit								
No	21 681	8928	77 643	11.5	1 (reference)		1 (reference)	
Yes	8758	5939	25 162	23.6	1.92 (1.86–1.98)	<0.001	1.46 (1.41–1.51)	<0.001

Abbreviations: IR, incidence rate; HR, hazard ratio; CI, confidence interval; BMI, body mass index.

^a^ Incidence per 1000 person-years.

^b^ Model 1 was not adjusted for any variables.

^c^ Model 2 was adjusted for all variables with p value of <0.05 in Model 1.

**Table 4:** Sex-specific risk scoring models for all-cause mortality in lung cancer.

	Male		Female	
	β regression coefficient	Point	β regression coefficient	Point
Age (years)				
19–44	1 (reference)		1 (reference)	
45–64	0.1012	8	0.0469	3
≥65	0.2929	24	0.2339	13
Income				
Others	1 (reference)		1 (reference)	
Low	0.0167	1	0.0315	2
Smoking status				
Non	1 (reference)		1 (reference)	
Current	0.0869	7	0.1303	7
Alcohol consumption				
No	1(Ref.)		1 (reference)	
Yes	0.0232	2	0.0231	1
Regular exercise				
None	1 (reference)		1 (reference)	
Regular	−0.051	−4	-0.0604	−3
BMI (kg/m^2^)				
<18.5	0.069	6	0.021	1
18.5–25	1 (reference)		1 (reference)	
≥25	−0.0366	−3	-0.0265	−1
Number of chronic diseases				
0	1 (reference)		1 (reference)	
1	0.012	1	0.0344	2
2	0.0293	2	0.0253	1
3	0.0429	4	0.0286	2
4	0.0516	4	0.0182	1
≥5	0.0762	6	0.0541	3
Treatment of lung cancer				
None	1 (reference)		1 (reference)	
Chemotherapy	0.1635	14	0.2853	16
Surgery	−0.4165	−35	−0.3518	−19
Radiotherapy	0.1302	11	0.2401	13
Surgery + chemotherapy	−0.2276	−19	−0.1632	−9
Surgery + radiotherapy	−0.1772	−15	−0.1168	−6
Chemotherapy + radiotherapy	0.1966	16	0.3417	19
Surgery + chemotherapy + radiotherapy	−0.1252	−10	−0.0589	−3
Emergence room visit				
No	1 (reference)		1 (reference)	
Yes	0.1187	10	0.1545	8

Abbreviations: BMI, body mass index.

### Risk of all-cause mortality according to risk categories in male and female patients

**Fig 1:**
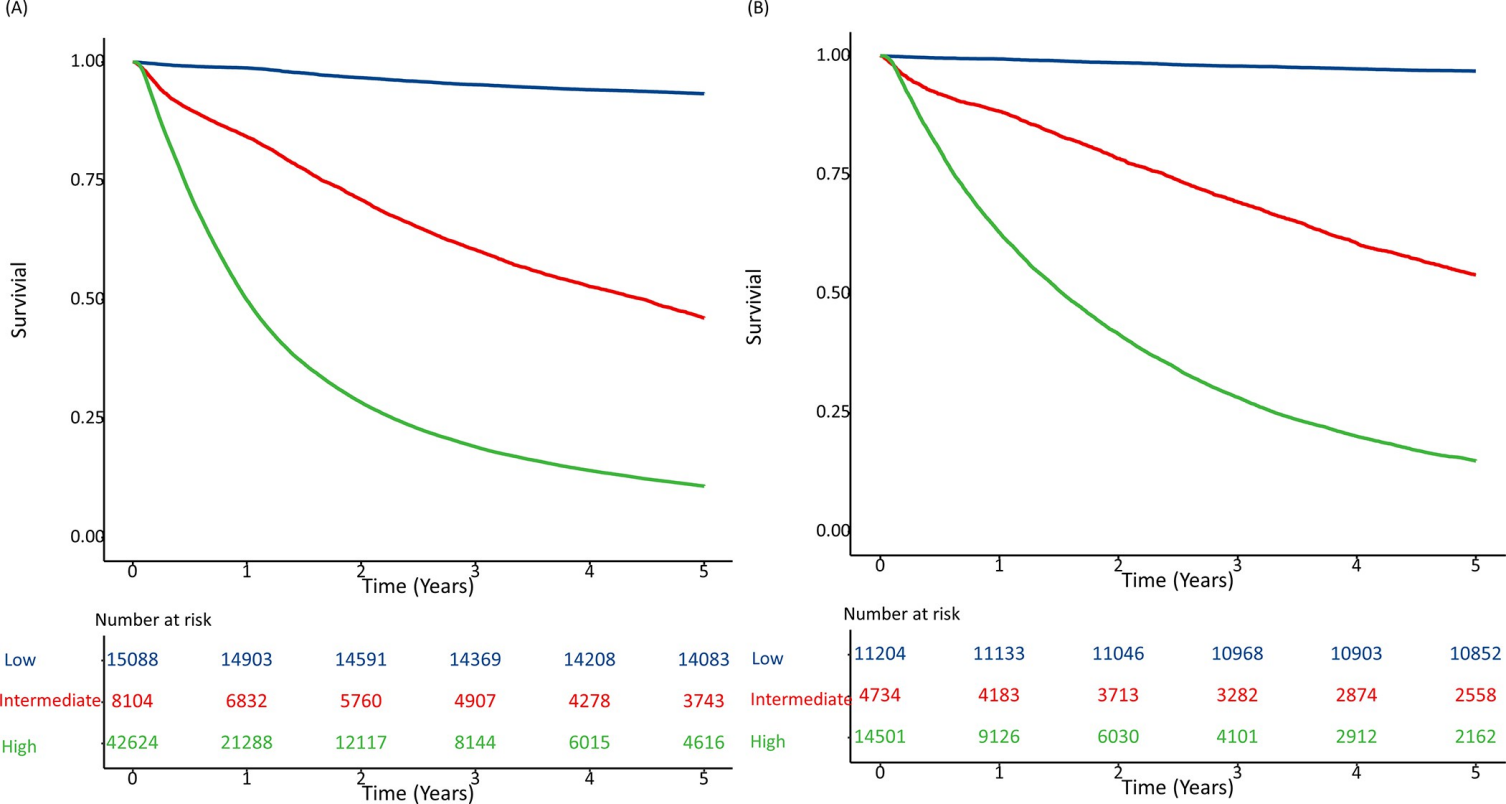
Kaplan–Meier survival curve according to risk level. (A) Male (B) Female.

Table 5 shows all-cause mortality risk at 1 and 3 years and within 5 years according to risk categories in male and female patients. In the overall cohort, 22.9%, 12.3%, and 64.8% of the male patients and 36.8%, 15.6%, and 47.6% of the female patients were in the low-, intermediate-, and high-risk groups, respectively. The mortality rates within 5 years were 7%, 54%, and 89% in the low-, intermediate-, and high-risk groups for male patients and 3%, 46%, 85% in the low-, intermediate-, and high-risk groups for female patients, respectively. Fig 1 shows the Kaplan-Meier curves according to the accumulation duration for risk category in male and female patients, and higher risk category had the probability of survival. The difference in the probability of 1-year mortality between the high- and low-risk groups was 0.49 for male patients and 0.36 for female patients. The c-statistics were 0.86 (95% CI, 0.85–0.87) and 0.85 (95% CI, 0.84–0.86) for male and female patients, respectively.

**Table 5:** Risk of all-cause mortality within 5 years and at 1 and 3 years in male and female patients according to risk category.

Sex	Risk category		Mortality within 5 years (%)	Mortality at 1 year	Mortality at 3 years
				% (95% CI)	% (95% CI)
Male	Low	15,088 (22.9)	7 (5–8)	1 (1–1)	5 (3–6)
	Intermediate	8104 (12.3)	54 (50–57)	16 (13–18)	39 (36–42)
	High	42,624 (64.8)	89 (88–90)	50 (49–51)	81 (80–82)
	Difference in probability ^a^		0.81	0.49	0.76
	c-statistic ^b^ (95% CI)		0.86 (0.85–0.87)		
Sex	Low	11,204 (36.8)	3 (2–4)	1 (0–2)	2 (2–2)
	Intermediate	4734 (15.6)	46 (42–50)	12 (10–13)	31 (26–35)
	High	14,501 (47.6)	85 (85–85)	37 (36–39)	72 (70–73)
	Difference in probability ^a^		0.82	0.36	0.70
	c-statistic ^b^ (95% CI)		0.85 (0.84–0.86)		

Abbreviations: CI, confidence interval.

^a^ Difference in the probability of mortality between the high- and low-risk groups was calculated using the following formula: (P_high_ − P_low_) / 100.

^b^ C-statistic for the overall score.

## Discussion

In the present study, we found sex disparities in several prognosis factors in a nationwide, large-scale retrospective cohort study of Korean patients with lung cancer. In addition, we developed novel sex-specific risk scoring models for prognostic prediction specifically in male and female patients based on these differences. Among the potential factors contributing to the prognosis of lung cancer, age ≥ 65 years was associated with the worst prognosis in male patients with lung cancer whereas chemotherapy in combination with radiotherapy was associated with the worst prognosis in female patients.

Many studies have evaluated sex differences associated with lung cancer prognosis. Biologic, hormonal, and genetic factors have been suggested as causes for the sex difference in lung cancer prognosis [15,16]. In one study, female patients with advanced lung cancer had significantly greater benefit from the addition of chemotherapy to anti-programmed death (PD)-1/PD ligand-1 treatment compared to male patients [17]. However, another study reported higher rates of immune-related side effects of anti-PD-1 therapeutics in female patients compared to male patients [18]. Therefore, it is likely that responses to anti-PD-1 treatment underlie the observed sex differences in lung cancer prognosis. Another study confirmed that estrogen-mediated suppression of inflammatory cytokine secretion by macrophages and neutrophils reduced the risk of cancer in females [19]. Yet another study found that sex differences in genetic susceptibility for lung cancer were due to polymorphisms in cytochrome P450 1A1 and glutathione S-transferase Mu 1 [20]. It is possible that these factors might have contributed to the differences in the risk scoring models developed to predict lung cancer mortality in female and male patients in the present study.

In Korea, the most common cause of death is malignant neoplasms and lung cancer is the most common cause of cancer-related deaths. The main cause of lung cancer is tobacco use, although the rate of current female smokers was very low in the current study cohort. Moreover, sex differences in the expression and mutation rate of some genes, such as K-ras, EGFR, and p53, might be a factor in sex prognostic differences [21,22]. These sex differences may be that female respond better to chemotherapy and radiation therapy for lung cancer, including small-cell lung cancer and non-small-cell lung cancer than male [23,24]. In particular, EGFR mutation is common in never-smoker Asian female [25,26] and previous study demonstrated that the effect of EGFR-tyrosine kinase inhibitor therapy of non-small-cell lung cancer was better response in female than male [27].

In the present study, female patients were younger than male patients, which might be associated with higher estrogen levels in these patients. In women, hormone replacement therapy and oral contraceptive use are associated with increased incidence of lung cancer, younger median age at lung cancer diagnosis, and shorter median survival time. Indeed, 39.1% of the male patients and 50.1% of the female patients were aged younger than 45 years in the present study (Tables 2 and 3). In addition, the number of women diagnosed with lung cancer before menopause is significantly higher than that of men diagnosed with lung cancer in the same age range [28]. Administration of estrogen preparations to reduce future heart disease in men as part of the Coronary Drug Project in the 1970s [29] was eventually stopped following an increase in lung cancer-related mortality.

In the present study, we also found sex differences in lung cancer treatment approaches. The rates of patients who did not receive treatment and those who received chemotherapy in combination with radiotherapy were higher in female patients than in male patients. These results suggest that women were diagnosed with more advanced or more aggressive disease compared to men. One possible reason for this phenomenon is that women were less likely to undergo screening for lung cancer than men given that the prevalence of smokers was lower in female patients than in male patients.

The present study has some limitations. First, we did not include lung cancer stage for categorization of patients in our analyses. However, we aimed to address this caveat by categorization according to the treatment methods. Second, not all modifiable variables were included and other factors that could affect the prognosis of lung cancer, such as nutritional status, smoking amount, alcohol consumption amount, and smoke or gas exposure during cooking, were not included. Third, various chemotherapy regimens are used for lung cancer, an aspect that was not considered in subgroup analyses in the present study. In addition, we did not include the stage of lung cancer because this information was available from the Korea NHIS. Fourth, the diagnosis of diseases based on ICD-10 codes might be underestimated or overestimated. Fifth, the study cohort included only patients from one city in Korea, which accounted for 16–22% of all Korean patients because of the lack of data and accessibility. Therefore, the extrapolation of the study findings to other ancestries and other cities of Korea might be limited.

However, the present study has several notable strengths that should be recognized. First, this was a large-scale study that utilized a cohort of adults and evaluated sex differences in lung cancer prognosis and associated factors. In addition, we developed novel, sex-specific risk scoring models to predict lung cancer prognosis in Korean patients, providing the basis for future, large-scale multinational studies to determine the utility of this approach.

In conclusion, the present study developed sex-specific risk scoring models to predict prognosis in patients with lung cancer. Utilization of these prognosis prediction models will aid in establishing nationwide policies, such as early cancer screen, education for smoking cessation, to tailor lung cancer treatment approaches according to sex, thereby improving the prognosis of lung cancer. Larger-scale worldwide studies are needed to develop sex-specific prognosis models for lung cancer.

## Abbreviations

BMI: Body mass index
CI: Confidence intervals
HR: Hazard ratio
ICD-10: International Classification of Diseases-10th
NHIS: National Health Insurance Service
